# Enhancer RNA commits osteogenesis via microRNA-3129 expression in human bone marrow-derived mesenchymal stem cells

**DOI:** 10.1186/s41232-022-00228-4

**Published:** 2022-09-16

**Authors:** Anh Phuong Nguyen, Kaoru Yamagata, Shigeru Iwata, Gulzhan Trimova, Tong Zhang, Yu Shan, Mai-Phuong Nguyen, Koshiro Sonomoto, Shingo Nakayamada, Shigeaki Kato, Yoshiya Tanaka

**Affiliations:** 1 The First Department of Internal Medicine, School of Medicine, University of Occupational and Environmental Health, Japan, 1-1 Iseigaoka, Yahata-nishi, Kitakyushu, 807-8555 Japan; 2 Endocrinology and Diabetes Department, Bach Mai Hospital, Hanoi, Vietnam; 3 Department of Clinical Subjects, High School of Medicine, Faculty of Medicine and Health Care, Al-Farabi Kazakh National University, Almaty, Kazakhstan; 4 Department of Basic Pathology, School of Medicine/School of Graduate Education, Fukushima Medical University, Fukushima, Japan; 5 Graduate School of Life Science and Engineering, Iryo Sosei University, Iwaki, Fukushima, Japan

**Keywords:** Super-enhancer, Enhancer RNA, miR-3129, Osteogenesis, Mesenchymal stem cells

## Abstract

**Background:**

Highly regulated gene expression program underlies osteogenesis of mesenchymal stem cells (MSCs), but the regulators in the program are not entirely identified. As enhancer RNAs (eRNAs) have recently emerged as a key regulator in gene expression, we assume a commitment of an eRNA in osteogenesis.

**Methods:**

We performed in silico analysis to identify potential osteogenic microRNA (miRNA) gene predicted to be regulated by super-enhancers (SEs). SE inhibitor treatment and eRNA knocking-down were used to confirm the regulational mechanism of eRNA. miRNA function in osteogenesis was elucidated by miR mimic and inhibitor transfection experiments.

**Results:**

miR-3129 was found to be located adjacent in a SE (osteoblast-specific SE_46171) specifically activated in osteoblasts by in silico analysis. A RT-quantitative PCR analysis of human bone marrow-derived MSC (hBMSC) cells showed that eRNA_2S was transcribed from the SE with the expression of miR-3129. Knockdown of eRNA_2S by locked nucleic acid as well as treatment of SE inhibitors JQ1 or THZ1 resulted in low miR-3129 levels. Overexpression of miR-3129 promoted hBMSC osteogenesis, while knockdown of miR-3129 inhibited hBMSC osteogenesis. Solute carrier family 7 member 11 (*SLC7A11*), encoding a bone formation suppressor, was upregulated following miR-3129-5p inhibition and identified as a target gene for miR-3129 during differentiation of hBMSCs into osteoblasts.

**Conclusions:**

miR-3129 expression is regulated by SEs via eRNA_2S and this miRNA promotes hBMSC differentiation into osteoblasts through downregulating the target gene *SLC7A11*. Thus, the present study uncovers a commitment of an eRNA via a miR-3129/*SLC7A11* regulatory pathway during osteogenesis of hBMSCs.

**Supplementary Information:**

The online version contains supplementary material available at 10.1186/s41232-022-00228-4.

## Background

Mesenchymal stem cells (MSCs) exist in limited organs and tissues, including the bone marrow, fat tissue, liver, muscle, and umbilical cord. MSCs exert anti-inflammatory and immunosuppressive activities through paracrine factors such as transforming growth factor β1 (TGF-β1) and exosomes [1–4]. They are also capable of self-renewal and mainly differentiate into mesodermal cell lineages, including adipocytes, chondrocytes, myoblasts, and osteoblasts [5, 6]. Their unidirectional differentiation is driven by various cytokines, growth factors, transcriptional factors, and microRNAs (miRNAs). Osteoblasts arise from mesenchymal precursors that undergo a highly regulated program of gene expression [7], but the regulators of the program are not entirely identified.

Super-enhancers (SEs) are a class of transcriptional regulatory regions that consist of multiple enhancers occupied by high densities of master transcription factors, co-activators, and mediator complexes [8]. SEs are spatially associated with the promoters of various genes, including both coding and non-coding genes like miRNAs that have important roles in determining cell identity. Enhancer RNAs (eRNAs), as SE modifiers, are transcribed from SE domains and subsequently interact with mediator and cohesin complexes to establish chromatin looping, which is essential for the interactions between enhancers and promoters and leads to the activation of target genes [9, 10]. The expression levels of eRNAs are correlated with the cis-regulatory activity of their SEs [11, 12]. Recent reports have revealed multiple roles of eRNAs in transcriptional regulation [13, 14]. However, little is known about the role of eRNAs in the transcription of miRNA genes.

miRNAs are a class of small non-coding RNAs that degrade messenger RNAs (mRNAs) and/or inhibit translation through post-transcriptional gene silencing. Some miRNAs, such as miR-138, miR-204, and miR-637, suppress osteogenic differentiation of MSCs [15–17]. Meanwhile, other miRNAs positively regulate osteogenesis. For example, Yang et al. [18] showed that miR-21 promoted osteogenic differentiation of bone marrow-derived MSCs by targeting hypoxia-inducible factor 1-*α* (*HIF-1α*) and vascular endothelial growth factor (*VEGF*) mRNAs in vitro. In some cases, SEs are located in the proximal regions of osteogenic genes and function as cis-regulatory elements for their expression, leading to dynamic osteogenesis of MSCs. However, the relationships between SEs/eRNAs and miRNAs during MSC osteogenesis remain to be fully clarified. In the present study, we focused on the regulatory mechanism of an osteogenic miRNA expression via SEs as well as the role of this miRNA in osteogenesis.

A group of miRNAs and a unique group of activated SEs that function in osteogenesis have been reported [19, 20]. As eRNAs have recently emerged as a key regulator in gene expression, therefore, we hypothesized that eRNAs transcribed from SEs regulate miRNA expression. The following experiments were conducted to elucidate the regulatory mechanism of miRNA expression by SEs activated in osteogenesis, using inhibitors of SE-forming factors and eRNA knockdown to inhibit SE function.

## Methods

### Cell culture

Human bone marrow-derived MSCs (hBMSCs) were purchased from Lonza (MD, USA) and used within 2–4 passages for the experiments. For these hBMSCs, cell surface markers, including CD29, CD44, CD105, and CD166, were positive, while CD14, CD34, and CD45 were negative as indicated by the manufacturer. The hBMSCs were cultured at 37°C under a 5% CO_2_ atmosphere in a MSC growth medium (PT-3001; MGM BulletKit; Lonza) and their confluence was maintained at <80% to prevent spontaneous differentiation.

### Primers and reagents

The primers were purchased from Thermo Fisher (MA, USA) for TaqMan-based qPCR primers and from Eurofins (Luxembourg) for SYBR Green-based qPCR primers. Primers to amplify eRNAs and LNA GapmeRs to knockdown eRNAs were designed using sequences in the dbSuper (https://asntech.org/dbsuper/; an integrated and interactive database of SEs [21]). Details of the eRNA primers and LNA GapmeRs are provided in Supplementary Tables S1 and S2 (see Additional files 1 and 2), respectively.

SLC7A11 antibody (ab238969) was purchased from Abcam (Cambridge, UK).

### Osteogenesis

Osteogenesis of hBMSCs was induced as described previously [22]. Briefly, hBMSCs at 80% confluence were trypsinized and seeded into Falcon® 24 well-plate purchased from Corning (Corning, USA) at 5×10^4^ cells/well. The cells were cultured in a commercial osteogenic induction medium (PT-3002; Lonza) for 21 days, with medium replacement every 3 days. Cell pellets were collected at specified time points for a series of analyses.

### Reverse transcription-quantitative polymerase chain reaction (RT-qPCR)

RNA extraction and RT-qPCR were performed as previously described [22]. Briefly, cultured cells were lysed in RLT buffer purchased from Qiagen (Hilden, Germany). Total RNA and miRNAs were extracted using RNeasy Mini Kit (Qiagen) and miRNeasy Mini Kit (Qiagen), respectively. For the total RNA, complementary DNAs (cDNAs) were synthesized using High-capacity cDNA Reverse Transcription Kit (Thermo Fisher) in accordance with the manufacturer’s instructions. For miRNAs, cDNAs were synthesized using a TaqMan MicroRNA Reverse Transcription Kit and amplified using TaqMan PreAmp Master Mix (Thermo Fisher). Real-time PCR was performed in a StepOnePlus system (Applied Biosystems, Thermo Fisher). The relative quantities of the transcripts were analyzed using the 2^−△△Ct^ method and normalized to the amounts of glyceraldehyde-3-phosphate dehydrogenase (*GAPDH*) for coding genes or *U6sn* for miRNA genes.

### Western blotting

Western blotting was carried out as described previously [23]. Briefly, MSC pellets or monolayer cells at indicated time points were sonicated (on/off cycle time: 30 s/30 s; cycle number: 5) in TNE lysis buffer containing 50mM Tris (pH 8.0), 150mM NaCl, and 1% Nonidet P40, supplemented with protease inhibitor cocktail (A-0014-20, ITSI Biosciences) and centrifuged at 12,000×g for 30 min at 4°C. The supernatant obtained after the centrifugation was used as whole-cell lysates (WCLs). WCL proteins (10 μg) were separated by 4–20% SDS-PAGE Tris-glycine gel and transferred onto a 0.2-μm nitrocellulose membrane (GE Healthcare). Immunoblotting was performed with primary antibodies followed by the appropriate secondary antibodies. β-actin was used as the loading control.

### Transfection of hBMSCs with pre-miR-3129-5p, miRVana miRNA mimic, and miRVana miRNA inhibitor

The following reagents were purchased from Thermo Fisher: Pre-miR™ miRNA Precursor (AM17100) of miR-3129-5p and its corresponding Pre-miR-negative control #1 (AM17110), miRVana miRNA mimic of miR-3129 (4464066) and its corresponding miRNA-negative control mimic #1 (4464058), and miRVana miRNA inhibitor of miR-3129 (4464084) and its corresponding miRNA-negative control inhibitor #1 (4464076). hBMSCs were seeded into 24-well plates at a density of 5×10^4^ cells/well in a MSC growth medium without antibiotics. The cells were transfected with transfection reagents comprising 5 pmol miRNA precursor and 1.5 μL Lipofectamine RNAiMAX (Invitrogen, Thermo Fisher) diluted in 100 μL Opti-MEM (31985062; Invitrogen), and then incubated at 37°C for 24h. Subsequently, the medium was replaced, and the cells were maintained in culture for further analyses. Transfection of hBMSCs with miRVana miRNA mimics (5 pmol) and inhibitors (5 pmol) was performed in accordance with the manufacturer’s instructions.

### Transfection of hBMSCs with LNA GapmeRs

Antisense LNA GapmeR Standard for potential eRNAs (339511) and its corresponding LNA-negative control B (339515) were purchased from Qiagen. hBMSCs were seeded into 24-well plates at a density of 1×10^5^ cells/well in a MSC growth medium without antibiotics. The cells were transfected with transfection reagents containing 30 pmol LNA GapmeR and 7 μL Lipofectamine RNAiMAX diluted in 100 μL OPTI-MEM, and then incubated at 37°C for 48h. Cell pellets were collected at specified time points for a series of analyses.

### Treatment of hBMSCs with SE inhibitors

JQ1 (BRD4 inhibitor; A1910) and THZ1 (CDK7 inhibitor; A8882) were purchased from APExBIO (TX, USA). hBMSCs were seeded into 24-well plates at a density of 5×10^4^ cells/well in a MSC growth medium without antibiotics. The cells were treated with JQ1 (60 and 250 nM) or THZ1 (50 and 100 nM) dissolved in DMSO for 24h. Cell pellets were collected at specified time points for a series of analyses.

### Alizarin Red S and alkaline phosphatase staining of hBMSCs

hBMSCs were cultured for 21 days to induce osteoblast differentiation [24]. The cells were fixed with 10% formalin at room temperature for 15 min. After three rinses with distilled water (DW), 300 μL of 2% Alizarin Red S (purchased from Sigma-Aldrich, MO, USA) was added to each well and incubated for 15 min. The cells were then rinsed three times with DW and observed under a microscope. Further staining to evaluate osteogenesis was performed using an Alkaline Phosphatase Staining Kit (PMC-AK20-COS; purchased from Cosmo Bio, Tokyo, Japan) in accordance with the manufacturer’s instructions. Both staining procedures were performed at day 3, 10, and 21 during osteogenesis, respectively. The results of the staining were quantified using ImageJ software as described previously [25]. Three independent experiments were performed.

### Statistical analysis

All quantitative data are expressed as mean ± SD for three independent experiments. Differences between two groups were tested for statistical significance by a Student’s unpaired two-tailed *t*-test. For comparisons of more than three groups, one-way analysis of variance (ANOVA) with a Bonferroni post hoc test was used. All statistical analyses were performed using IBM SPSS Statistics version 26.0 software (IBM Corp., USA). Values of *p* < 0.05 were considered significant.

## Results

### eRNAs from super-enhancers located near the miR-3129 gene region are highly expressed in the early stage of hBMSC osteogenesis

**Fig. 1:**
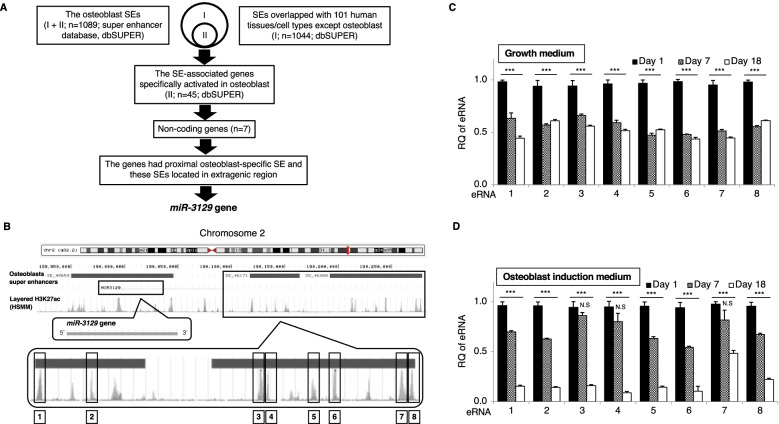
The *miR-3129* gene located nearby osteoblast-specific SE domains and relevant eRNA expression. **A** Schematic diagram of the in silico analysis, based on the dbSUPER database. **B** Two osteoblast-specific SE (SE_46171 and SE_46098) domains are located near the *miR-3129* gene on chromosome 2, and eight putative eRNAs may be expressed around H3K27ac. **C**, **D** Eight types of eRNA were highly expressed during the early stage of MSC differentiation into osteoblasts. The levels of eRNA expression were quantified by qPCR. The amounts of the eRNA transcripts were expressed relative to the amount of *GAPDH* transcript. Data are expressed as mean ± SD from three independent experiments (each *n* = 3 in **C** and **D**). ***p<0.001 vs. day 1 by one-way ANOVA*.* N. S, not significant; RQ, relative quantification

SEs have been reported to determine cell identity by inducing cell differentiation [8]. To identify novel SE-associated genes that can induce osteoblast differentiation, we performed in silico mining to search for potential osteogenic genes regulated by SEs that are specifically activated in osteoblasts (Fig. 1A). Firstly, we focused on 1089 genes regulated by SE activated in osteoblasts through dbSuper. Next, we extracted many genes activated in 101 human tissues/cell types except osteoblasts, screened 1044 genes overlapped with osteoblast, and excluded 1044 common genes of 1089 genes activated in osteoblast. As a result, we found 45 genes (including 7 non-coding RNAs) specifically regulated by SE in osteoblasts (Supplementary Table S3 (see Additional file 3)). Our interest was to study the regulatory mechanism and role of a non-coding RNA which remains to be defined. Therefore, we attempted to find a candidate non-coding gene which had proximal and extragenic (gene-free) SEs. Finally, miR-3129 was extracted as a candidate gene. The miR-3129 locus has a SE structure (SE_45650 registered in dbSUPER) located in an intragenic region and two SEs (SE_46171 and SE_46098) located in the proximal extragenic region (Fig. 1B). Within these regions, there are several peaks for histone H3 containing acetylated lysine 27 (H3K27ac), a SE marker, in human skeletal muscle myoblasts. We picked up 8 regions of the registered high H3K27ac peaks on the database and assumed these regions transcribe eRNAs in these 2 SE domains, given that eRNAs are defined as non-coding RNAs transcribed from SE domains encompassing layer(s). Therefore, we performed RT-qPCR and then found at least 8 types of eRNAs in 8 different regions within SE_46171 and SE_46098 in the hBMSCs cultured with a growth medium and an osteoblast induction medium (Fig. 1C, D). The hBMSCs that have just adhered to the plate might be unstable in gene expression profiles, presumably showing higher expression in the case of eRNAs. These eRNAs were most highly expressed in the SE domains during the early stage of osteoblast differentiation, and their expression gradually decreased as differentiation progressed (Fig. 1D).

### eRNAs and miR-3129 expression were suppressed by SE inhibitor treatment in a time-dependent and dose-dependent manner

**Fig. 2:**
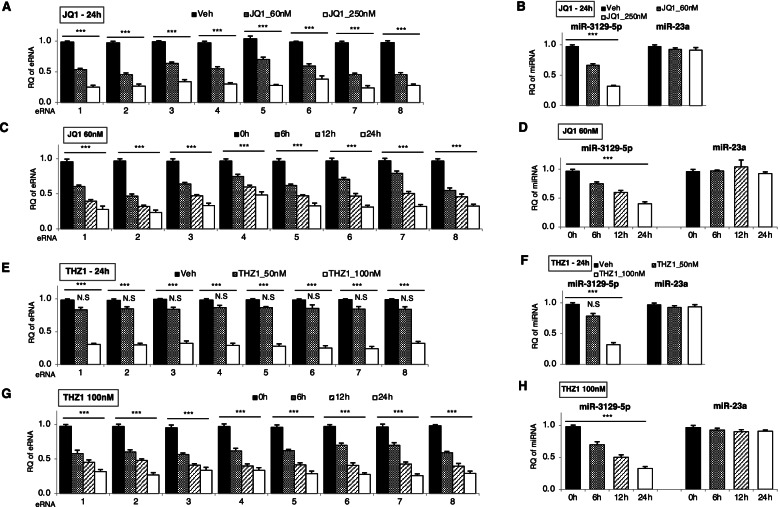
miR-3129-5p expression was suppressed by SE inhibitors in a time-dependent and dose-dependent manner. **A**, **C**, **E**, **G** eRNA expression was examined at 24h after treatment of MSCs with the indicated concentrations of JQ1 (**A**) and THZ1 (**E**) and at the indicated time points after treatment of MSCs with 60 nM JQ1 (**C**) and 100 nM THZ1 (**G**). **B**, **D**, **F**, **H** Expression of miR-3129-5p and miR-23a was examined at 24h after treatment of MSCs with the indicated concentrations of JQ1 (**B**) and THZ1 (**F**) and at the indicated time points after treatment of MSCs with 60 nM JQ1 (**D**) and 100 nM THZ1 (**H**). The levels of eRNA and miRNA expression were quantified by qPCR. The amounts of eRNA and miRNA transcripts were expressed relative to the amounts of *GAPDH* and *U6sn* transcripts, respectively. Data are expressed as mean ± SD from three independent experiments (each *n* = 3 in **A**–**H**). ***p<0.001 vs. Veh or 0 h by one-way ANOVA. N.S, not significant; RQ, relative quantification; Veh, vehicle (DMSO)

In order to determine the involvement of eRNAs in miR-3129 expression, we attempted to suppress eRNA level by SE inhibitors and then the effect of eRNA on miR-3129 expression. The transcriptional co-activators bromodomain-containing protein 4 (BRD4) and cyclin-dependent kinase 7 (CDK7), which are responsible for SE formation, are abundantly localized in SEs which express eRNAs. Therefore, we focused on JQ1 and THZ1 as SE inhibitors, which specifically inhibit BRD4 and CDK7 activity, respectively, and then examined the effect of SE inhibitors on miR-3129 levels as well as eRNA levels. At first, hBMSCs were stimulated with various concentrations of JQ1 (60 nM and 250 nM) for 24h. The eRNAs and miR-3129 expression were then determined by RT-qPCR. SEs are generally cell-type-specific and can function as cis-regulatory elements for nearby genes. Therefore, miR-23a, a miRNA highly expressed in the early stage of hBMSC osteogenesis [26], was used as a control miRNA for miR-3129 in these experiments. As a result, the expression of each eRNA was significantly suppressed in a dose-dependent manner (Fig. 2A). The expression of miR-3129, but not miR-23a, was also suppressed (left side and right side of Fig. 2B). Next, hBMSCs were stimulated with JQ1 at a concentration of 60 nM for specified time points (0h, 6h, 12h, and 24h). The expression of each eRNA (Fig. 2C) as well as miR-3129, but not miR-23a (left side and right side of Fig. 2D), was significantly suppressed in a time-dependent manner (Fig. 2C, D). Similarly, hBMSCs were stimulated with various concentrations of THZ1 (50 nM and 100 nM) for 24h, and the expression of each eRNA (Fig. 2E) as well as miR-3129, but not miR-23a (left side and right side of Fig. 2F), was significantly suppressed in a dose-dependent manner. Furthermore, when hBMSCs were stimulated with THZ1 at a concentration of 100 nM for specified time points (0h, 6h, 12h, and 24h), the expression of each eRNA (Fig. 2G) as well as miR-3129, but not miR-23a (left side and right side of Fig. 2H), was suppressed in a time-dependent manner. These results indicated that miR-3129 expression was suppressed by SE inhibitor treatment in a time-dependent and dose-dependent manner.

### Knockdown of eRNA_2S resulted in suppressing miR-3129 expression

**Fig. 3:**
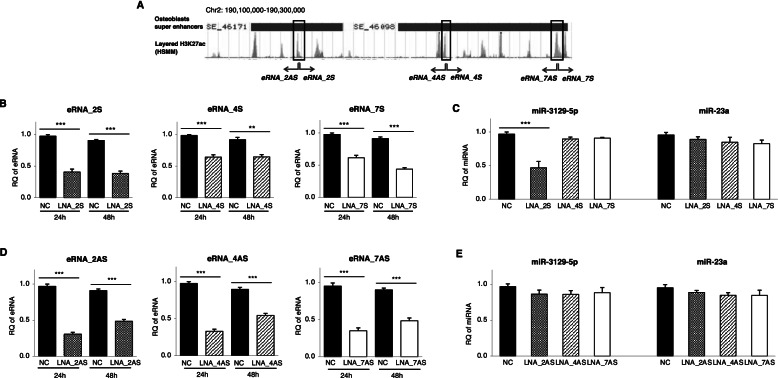
Knockdown of eRNA_2S suppressed miR-3129-5p expression in hBMSCs. **A** Positions of the putative eRNA_2, eRNA_4, and eRNA_7 transcripts during MSC differentiation into osteoblasts. **B**, **D** Each sense (**B**) and antisense (**D**) eRNA was examined in MSCs transfected with antisense LNA GapmeR. **C**, **E** miR-3129-5p expression was examined in MSCs transfected with sense (**C**) and antisense (**E**) LNA GapmeRs for 24h. The expression of miR-23a was examined as a control. The levels of eRNA and miRNA expression were quantified by qPCR. The amounts of eRNA and miRNA transcripts were expressed relative to the amounts of *GAPDH* and *U6sn* transcripts, respectively. Data are expressed as mean ± SD from three independent experiments (each *n* = 3 in **B**, **C**, **D**, and **E**). **p = 0.001, ***p < 0.001 vs. NC by Student’s *t*-test and one-way ANOVA. LNA, locked nucleic acid; NC, LNA GapmeR-negative control; S, sense; AS, antisense; RQ, relative quantification

In general, eRNAs play roles in the induction of their target gene expression. Therefore, we examined the expression of miR-3129 after eRNA knockdown. We randomly chose eRNA 2, 4, and 7 as the candidate eRNAs to investigate the roles of individual eRNAs including sense (S) and antisense (AS) eRNAs (Fig. 3A). Locked nucleic acid (LNA) sequences designed to target each eRNA and a LNA-negative control (LNA-NC) sequence were transfected into hBMSCs (Supplementary Table S2 (see Additional file 2)). The knockdown efficiency of various sense eRNAs and miR-3129 expression were examined by RT-qPCR (Fig. 3B). The expression of miR-3129 was only significantly decreased in MSCs after knockdown of eRNA_2S (transcribed from SE_46171) (Fig. 3C). The knockdown efficiency of various antisense eRNAs was also elucidated (Fig. 3D). However, the expression of miR-3129 was not altered after knockdown of any antisense eRNAs (Fig. 3E). Collectively, only knockdown of eRNA_2S showed an effective impact on miR-3129 expression.

### miR-3129 expression was downregulated during osteoblast differentiation and pre-miR-3129 precursor transfection enhanced hBMSC osteogenesis

**Fig. 4:**
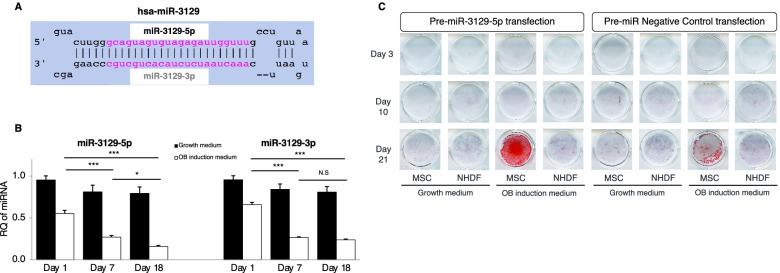
miR-3129 was expressed in the early stage of osteoblast differentiation and enhanced osteogenesis of hBMSCs. **A** Sequences and structure of human hsa-miR-3129, based on the miRBase database. **B** miR-3129 expression was examined at the indicated time points during osteogenesis of MSCs. The levels of miRNA expression were quantified by qPCR. The amounts of miRNA transcripts were expressed relative to the amount of *U6sn* transcript. Data are expressed as mean ± SD from three independent experiments. **C** Calcification was examined in MSCs and NHDF cells treated with Pre-miR-3129-5p and Pre-miR-NC by Alizarin Red S staining. Representative data are provided from three independent experiments (each *n* = 3 in **B** and **C**). *p<0.05, ***p<0.001 vs. day 1 by one-way ANOVA. N.S, not significant; RQ, relative quantification; OB, osteoblast. NHDF, normal human dermal fibroblast

The secondary structure of the human hsa-miR-3129 precursor from miRBase (a miRNA database [27]) is shown in Fig. 4A. The expression pattern of miR-3129 during hBMSC differentiation into osteoblasts was examined by RT-qPCR. hBMSCs expressed both the 5p and 3p strands of miR-3129 regardless of the cell proliferation time (day 1, 7, and 18), and the expression levels remained unchanged (Fig. 4B). When hBMSCs were cultured in an osteoblast induction medium (OIM), the 5p and 3p strands both showed the highest expression during the early stage (day 1), and their expression gradually decreased as osteogenesis progressed (day 7 and 18). As controls for OIM, hBMSCs were cultured in adipocyte and chondrocyte induction media. The results show that expression levels of the 5p and 3p strands remained unaltered during the respective differentiation processes (Supplementary Fig. S1 (see Additional file 4)), indicating the specific role of miR-3129 in osteogenesis.

We transfected hBMSCs with a Pre-miRNA-3129 precursor and a Pre-miRNA-negative control precursor (Pre-miR-NC) to further clarify the miR-3129 function in the osteoblast differentiation process. In OIM culture, the cells showed positive Alizarin Red S (ARS) staining on day 21 (Fig. 4C). Normal human dermal fibroblast (NHDF) cells, as a control cell line, were also transfected with these precursors. Interestingly, hBMSCs transfected with the Pre-miR-3129 precursor showed stronger positivity on day 21 compared with those transfected with the Pre-miR-NC. NHDF cells were negative for ARS staining regardless of the culture medium and regardless of which miRNA precursor was transfected.

### miR-3129 overexpression promoted osteoblast differentiation of hBMSCs through downregulating SLC7A11

**Fig. 5:**
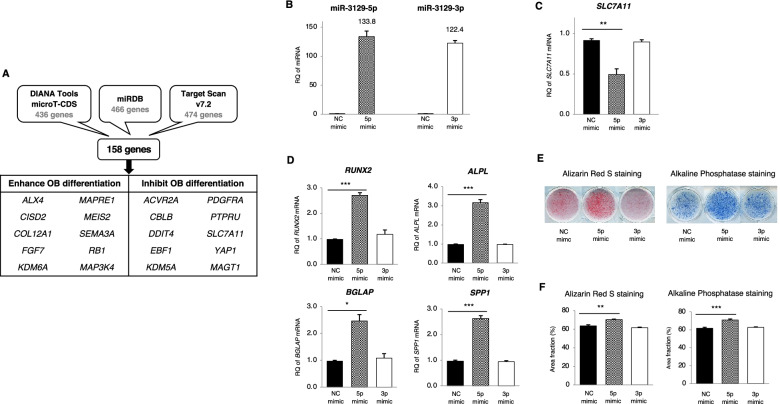
miR-3129-5p mimic transfection enhanced hBMSC osteoblast differentiation through downregulating *SLC7A11*. **A** Massive screening for miR-3129-5p putative target genes using three different databases. **B**–**D** Expression of miR-3129 (**B**), *SLC7A11* (**C**), and osteoblast markers (**D**) in MSCs at 48h (**B**, **C**) and day 18 (**D**) after transfection with miR-3129-5p and miR-3129-3p mimics. The levels of miRNA and mRNA expression were quantified by qPCR. The amounts of miRNA and mRNA transcripts were expressed relative to the amounts of *U6sn* and *GAPDH* transcripts, respectively. **E**, **F** Alizarin Red S and alkaline phosphatase staining were performed in three independent experiments at day 21 after transfection of miR-3129 mimics (**E**), followed by quantification with ImageJ software (**F**). Data are expressed as mean ± SD from three independent experiments (each *n*=3 in **B**, **C**, **D**, **E**, and **F**). *p < 0.05, **p = 0.001, ***p < 0.001 vs. NC mimic by Student’s *t*-test. NC, negative control; OB, osteoblast; RQ, relative quantification

Using three public databases (DIANA Tools microT-CDS, miRDB, and Target Scan v7.2), we extracted three groups of genes predicted to be the target of this miRNA (Fig. 5A). As a result of in silico analyses, 158 overlapping genes were found. We further divided them into 10 genes that promote osteogenesis and 10 genes that suppress osteoblast differentiation. Our previous results showed that miR-3129 has the potency to enhance osteoblast differentiation (Fig. 4C); therefore, finding a target for this miRNA will be focused on 10 genes that are suppressors of osteogenesis. hBMSCs were transfected with the miR-NC mimic, miR-3129-5p mimic, and miR-3129-3p mimic, and high levels of the 5p and 3p strands were detected, respectively, after 48h (Fig. 5B). Compared with transfection of the miR-NC mimic, transfection of the 5p mimic only decreased the expression of solute carrier family 7 member 11 (*SLC7A11*) among the 10 osteogenic suppressor genes (Fig. 5A), while transfection of the 3p mimic did not affect the expression of any candidate genes in mRNA levels (Fig. 5C and Supplementary Fig. S2A (see Additional file 5)). The expression of osteoblast transcription factor and marker genes such as runt-related transcription factor 2 (*RUNX2*), alkaline phosphatase gene (*ALPL*), bone gamma-carboxyglutamate protein (*BGLAP*), and secrected phosphoprotein 1 (*SPP1*) were significantly increased by the transfection of the 5p mimic, but not the miR-NC mimic and 3p mimic (Fig. 5D). ARS and alkaline phosphatase (ALP) staining were performed to evaluate the phenotype after osteogenesis (Fig. 5E). Transfection of the miR-NC mimic and 3p mimic resulted in similar levels of positive staining for ARS and ALP, whereas transfection of the 5p mimic significantly increased both staining levels (Fig. 5F).

### Knockdown of miR-3129 resulted in suppressed osteoblast differentiation of hBMSCs

**Fig. 6:**
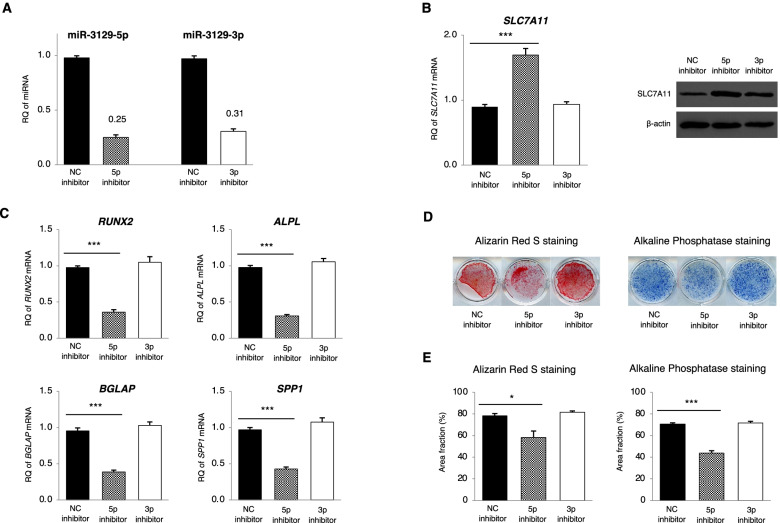
Inhibition of miR-3129-5p attenuated osteogenesis of hBMSCs. **A**–**C** Expression of miR-3129 (**A**), SLC7A11 mRNA and protein (**B**), and osteoblast markers (**C**) in MSCs at 48h (**A, B**) and day 18 (**C**) after transfection of miR-3129-5p and miR-3129-3p inhibitors. The levels of miRNA and mRNA expression were quantified by qPCR. The amounts of miRNA and mRNA transcripts were expressed relative to the amounts of *U6sn* and *GAPDH* transcripts, respectively. **B** The levels of SLC7A11 protein were detected by Western blotting. β-actin was used as a loading control. The images cropped from the data (*n*=1) on Fig. S4 are shown on the right side of Fig. 6B (see Additional file 7). **D**, **E** Alizarin Red S and alkaline phosphatase staining were performed in three independent experiments on day 21 after transfection of miRNA inhibitors (**D**), followed by quantification with ImageJ software (**E**). Western blotting was performed one time (*n* = 1 in **B**). Data are expressed as mean ± SD from three independent experiments (each *n* = 3 in **A**–**H**). *p<0.05, ***p<0.001 vs. NC inhibitor by Student’s *t*-test. NC, negative control; RQ, relative quantification

Next, we evaluated the effect of knocking down the 5p and 3p strands of miR-3129 on hBMSC osteoblast differentiation with specific inhibitors. At 48h after transfection of hBMSCs with the miR-NC, miR-5p, and miR-3p inhibitors, we found that the expression of the 5p and 3p strands was suppressed (Fig. 6A). Compared with hBMSCs treated with the miR-NC inhibitor, those treated with the 5p inhibitor displayed an upregulated expression of only SLC7A11 mRNA and protein, but not mRNAs of other osteogenic suppressor genes (Fig. 5A, Fig. 6B, and Supplementary Fig. S2B (see Additional file 5)). In contrast, those treated with the 3p inhibitor showed no such upregulation in SLC7A11 mRNA and protein level (Fig. 6B) as well as mRNAs of other osteogenic suppressor genes (Fig. 5A and Supplementary Fig. S2B (see Additional file 5)). Meanwhile, there were no differences in the expression levels of a series of osteoblast transcription factor and marker genes between the miR-NC inhibitor and 3p inhibitor, while the 5p inhibitor significantly suppressed the expression of these genes (Fig. 6C). Staining for ARS as well as ALP was performed to evaluate the phenotype after osteogenesis (Fig. 6D). Treatment with the miR-NC and 3p inhibitors resulted in similar levels of positive staining for ARS and ALP, while treatment with the 5p strand inhibitor significantly suppressed both staining levels (Fig. 6E).

### SLC7A11 expression enhanced after eRNA_2S knockdown in hBMSCs

In our previous results, *SLC7A11* was identified as the miR-3129 target gene in hBMSCs (Figs. 5 and 6). We further examined the involvement of eRNAs in the regulation of *SLC7A11* expression and found that only knockdown of eRNA_2S significantly increased the *SLC7A11* level (Supplementary Fig. S3 (see Additional file 6)). This result was consistent with our previous findings and further determined the regulatory role of eRNA_2S in miR-3129 expression in hBMSCs.

## Discussion

Through a massive in silico approach, *miR-3129* was extracted as a candidate gene that is specifically regulated by SEs in osteoblasts. Subsequently, the miR-3129 expression level was confirmed to be high during the early stage of osteogenesis of hBMSCs (Fig. 1). Given that the *miR-3129* gene specifically forms SE structures in osteoblasts, an important regulatory role of SEs in miR-3129 expression was expected during the early stage of osteogenesis. In fact, SE inhibitors reduced the levels of eRNAs and miR-3129 expression from the SEs (Fig. 2). In particular, knockdown of eRNA_2S alone reduced the levels of miR-3129 expression (Fig. 3). Therefore, critical involvement of SEs in miR-3129 induction was expected.

MSCs differentiate into mesodermal cell types, including osteoblasts. During this process, dramatic changes in the expression of a genome-wide set of genes are responsible for individual cell differentiation [7]. Recently, it was reported that SEs activate their target genes, promote cell differentiation, and define cell identity [8]. In fact, activation of SEs promotes chondrocyte and adipocyte differentiation [28, 29]. Furthermore, when examining the peak of H3K27ac (SE marker) by chromatin immunoprecipitation sequencing (ChIP-seq) analysis, 1680 SE domains were found in hBMSCs [19]. Among them, 1380 SEs were unique for hBMSCs and 42 SEs were unique for osteoblast differentiation (day 14). As a result, 300 SEs were common in both groups. In other words, multiple SEs in hBMSCs regulate the expression of target genes that define osteogenesis. In the future, it will be interesting to determine whether miR-3129 is regulated by common SEs or unique SEs.

Two SE domains (SE_46171 and SE_46098) exist in the proximal region of the *miR-3129* gene. Based on our findings, eRNAs were expressed from these domains in hBMSCs and during osteoblast differentiation. Among the group of multiple eRNAs, knockdown of eRNA_2S (transcribed from SE_46171) decreased miR-3129-5p expression. eRNA_2S is predicted to induce miR-3129 expression at the promoter level. In general, eRNAs play crucial roles in transcriptional activation through the following events: (i) recruitment of transcription factors to promoters and enhancers of target genes, (ii) drastic looping between SEs and promoters, (iii) chromatin remodeling, (iv) RNAP II activation by recruitment to target promoters or phosphorylation, (v) histone tail acetylation, and (vi) regulation of liquid–liquid phase separation [13, 14]. Elucidation of the detailed molecular mechanism for how eRNA_2S is responsible for the regulation of *miR-3129* gene expression remains a challenge for future studies.

Previously, miR-3129 was reported to regulate retinoblastoma protein (pRb) to promote the viability of the gastric cancer cell line SGC7901 [30], but other functions of miR-3129 remain unknown. After transfection of hBMSCs with miR-3129-5p mimics (Fig. 5) and inhibitors (Fig. 6) and culture of the cells with OIM, ARS staining was denser and fainter, respectively, compared with that in the control group. These findings emphasize that miR-3129 enhances the osteogenesis of hBMSCs. In silico analyses using a set of miRNA target prediction programs were performed to investigate the molecular mechanism for osteogenesis (Fig. 5A). Transfection of the mimic and inhibitor for miR-3129-5p, but not miR-3129-3p, significantly reduced and increased the amount of *SLC7A11* mRNA expression, respectively (Fig. 5C, Fig. 6). Transfection of the inhibitor for miR-3129-5p, but not miR-3129-3p, also upregulated SLC7A11 protein, respectively (Fig. 6B and Supplementary Fig. S4 (see Additional file 7)). Importantly, miR-3129 is predicted to bind to different sites (Chr 4: 139092489-139092511; 139089616-139089642; 139087705-139087722; 139086969-139086987; 139085552-139085563) of SLC7A11 mRNA based on microT-CDS (DIANA Tools). Previous reports have shown that the SLC7A11 protein is localized on the plasma membrane and acts as a suppressor of osteoblast differentiation by increasing downstream glutathione via the glutamate/cystine antiporter [31, 32]. Therefore, we found that miR-3129-5p promotes hBMSC differentiation into osteoblasts through downregulating SLC7A11, which is involved in cell metabolism. A limitation of our study is that we were unable to clarify the extent to which miR-3129 is expressed in MSCs derived from rheumatoid arthritis (RA) patients and whether it is actually responsible for osteoblast differentiation. Further studies on MSCs from RA patients will reveal the expression of miR-3129 in these cells and during their osteogenesis, to highlight the role of miR-3129 as a disease-independent function.

In silico analyses revealed that miR-3129 may be regulated by SEs that are specifically activated in osteoblasts. We also found a subset of eRNAs expressed early in osteogenesis that regulated miR-3129 expression via SE activation. The Pre-miR-3129 precursor promoted differentiation of hBMSCs into osteoblasts. A group of 20 genes involved in osteogenesis were identified as possible targets of miR-3129 by multiple prediction programs. After transfection of the miR-3129 mimics and inhibitors into hBMSCs, we focused on the *SLC7A11* gene encoding a cystine/glutamate antiporter as a novel target gene for miR-3129 in osteogenesis. Furthermore, knockdown of eRNA_2S transcribed from the SE_46171 near the *miR-3129* gene not only decreased the expression of miR-3129 but also increased the expression of SLC7A11. The results indicated that eRNA_2S might regulate miR-3129 expression, followed by suppressing *SLC7A11* level in hBMSCs to promote their differentiation into osteoblasts (Supplementary Fig. S4 (see Additional file 7)). These findings suggest that miR-3129 is involved in hBMSC osteogenesis. This is the first report that eRNA_2S, transcribed from osteoblast-specific SE_46171, is involved in the regulation of miR-3129 expression.

## Conclusion

In summary, we demonstrate the regulatory involvement of eRNA in an osteogenic miRNA expression. Our study unveils that knockdown of eRNA_2S (transcribed from osteoblast-specific SE_46171) results in suppressing miR-3129 level. In addition, this miRNA can enhance hBMSC osteoblast differentiation through downregulating *SLC7A11*. These findings suggest that the eRNA_2S/miR-3129/SLC7A11 axis has an important role and may be a potential target for the regulation of hBMSC osteogenesis.

## Supplementary Information

**Additional file 1: Supplementary Table S1.** Sequences of the primers used to detect eRNAs.

**Additional file 2: Supplementary Table S2.** LNA GapmeR sequences designed to knock-down eRNAs.

**Additional file 3: Supplementary Table S3.** List of 45 genes predicted to be regulated by osteoblast-specific SEs based on the dbSUPER database.

**Additional file 4: Supplementary Figure S1.** *miR-3129* gene expression pattern during adipogenesis and chondrogenesis of hBMSCs. Expression of miR-3129 was examined on Day 1, 7, 14, and 21 during adipogenesis **(A)** and chondrogenesis **(B)** of hBMSCs. The levels of miRNA expression were quantified by qPCR. The amounts of the miRNA transcript was expressed relative to the amount of *U6sn* transcript. Data are expressed as mean ± SD from three independent experiments (each n=3 in **A** and **B**).

**Additional file 5: Supplementary Figure S2.** Expression profiling of other putative target genes for miR-3129-5p after overexpression and knock-down of miR-3129 in hBMSCs. **(A and B)** At 48h after transfection of miR-3129-5p and -3p mimics **(A)** and -5p and -3p inhibitors **(B)**, the levels of miR-3129-5p putative target gene expression were quantified by qPCR. The amounts of the mRNA transcript were expressed relative to the amount of *GAPDH* transcript. Data are expressed as mean ± SD from three independent experiments (each n=3 in **A** and **B**). m, mimic; in, inhibitor; NC, negative control.

**Additional file 6: Supplementary Figure S3.** Knock-down of eRNA_2S enhanced *SLC7A11* gene expression in hBMSCs. **(A and B)** At 24h after transfection of the indicated sense **(A)** and antisense **(B)** LNA GapmeRs, the levels of *SLC7A11* expression were quantified by qPCR. The amounts of the *SLC7A11* transcript were expressed relative to the amount of *GAPDH* transcript. Data are expressed as mean ± SD from three independent experiments (each n=3 in **A** and **B**). ***p<0.001 versus NC, by Student’s *t*-test. LNA, Locked Nucleic Acid; S, sense; AS, antisense; NC, LNA negative control.

**Additional file 7: Supplementary Figure S4.** All full-length images of Western blotting data. Uncropped full-length images are shown on Figure S4, and the images cropped from the data (n=1) on the Figure S4 are shown on the right side of Fig. 6B. Western blotting was performed one time.

**Additional file 8: Supplementary Figure S5.** A model of the expression mechanism for miR-3129 involved in MSC osteogenesis. An eRNA is expressed during the early stage of MSC osteoblast differentiation, and then induces miR-3129 expression via SE activation. The miRNA targets the mRNA of *SLC7A11*, which encodes a suppressor of osteogenesis, followed by down-regulation of *SLC7A11* expression. This event contributes to MSC osteogenesis. BMSC, bone marrow-derived mesenchymal stem cell; RNA Pol II, RNA polymerase II; SE, super-enhancer; TF, transcription factor; TSS, transcription start site.

## Data Availability

The dataset supporting the conclusions of this article is available in the Dryad Digital Repository, doi:10.5061/dryad.x69p8czm7 [33].

## Abbreviations

MSC: Mesenchymal stem cell
hBMSC: Human bone marrow-derived mesenchymal stem cell
dbSUPER: Database of super-enhancers
SE: Super-enhancer
eRNA: Enhancer RNA
miRNA: MicroRNA
mRNA: Messenger RNA
cDNA: Complementary DNA
ChIP-seq: Chromatin immunoprecipitation sequencing
LNA: Locked nucleic acid
S: Sense
AS: Antisense
WCLs: Whole-cell lysates
H3K27ac: Histone H3 containing acetylated lysine 27
BRD4: Bromodomain-containing protein 4
CDK7: Cyclin-dependent kinase 7
SLC7A11: Solute carrier family 7 member 11
TGF-β1: Transforming growth factor beta 1
HIF-1α: Hypoxia-inducible factor 1 subunit alpha
VEGF: Vascular endothelial growth factor
RUNX2: Runt-related transcription factor 2
ALPL: Alkaline phosphatase gene
BGLAP: Bone gamma-carboxyglutamate protein (osteocalcin)
SPP1: Secreted phosphoprotein 1 (osteopontin)
GAPDH: Glyceraldehyde-3-phosphate dehydrogenase
pRb: Retinoblastoma protein
ARS: Alizarin Red S
ALP: Alkaline phosphatase
OB: Osteoblast
OIM: Osteoblast induction medium
NHDF: Normal human dermal fibroblast
RA: Rheumatoid arthritis
DW: Distilled water
NC: Negative control
RQ: Relative quantification
