# Supplementary material for: Enhancer RNA commits osteogenesis via microRNA-3129 expression in human bone marrow-derived mesenchymal stem cells
Source: Inflamm Regen. 2022 Sep 16;42:43. doi: 10.1186/s41232-022-00228-4 (PMC9479228; doi:10.1186/s41232-022-00228-4)
Supplement: Supplementary file 1 — Additional file 1: Supplementary Table S1. Sequences of the primers used to detect eRNAs. [file 41232_2022_228_MOESM1_ESM.pdf]

Additional file 1

Supplementary Table S1. Sequences of the primers used to detect eRNAs

**eRNA**	**Sequence**
eRNA_1	(FW) CAGCACAACCGTCCTGACT<br>(RV) TGTCTTTTGGCCCCTGGT
eRNA_2	(FW) TTCTCCAAATTAGGCGGTGT<br>(RV) TGACATTAAATAGCATGTGGTGGT
eRNA_3	(FW) ATCTTGCAGTTGTTAGCAAGCAT<br>(RV) TGCTGTAAATTTTGGGTTGG
eRNA_4	(FW) CTGCACTGCCACAGGATG<br>(RV) GGAATCTCCAGAGAGTGAAGGA
eRNA_5	(FW) TTCTCCGTCATCTCATCTCTACC<br>(RV) TTGCTCTTTCAGCTTTACCACA
eRNA_6	(FW) AAGCAACCAAGGCTCAGAGA<br>(RV) AGAAAGACCACGAGCTTGGA
eRNA_7	(FW) GACCTCGTTATGGCAAGTGA<br>(RV) CCAGGTAAGCAGAGCCAGTC
eRNA_8	(FW) AATATCCTGTGAGCCGCAGT<br>(RV) CTTTCTCACTGTCTTTTCCCTCTC
