# Supplementary material for: Enhancer RNA commits osteogenesis via microRNA-3129 expression in human bone marrow-derived mesenchymal stem cells
Source: Inflamm Regen. 2022 Sep 16;42:43. doi: 10.1186/s41232-022-00228-4 (PMC9479228; doi:10.1186/s41232-022-00228-4)
Supplement: Supplementary file 2 — Additional file 2: Supplementary Table S2. LNA GapmeR sequences designed to knock-down eRNAs. [file 41232_2022_228_MOESM2_ESM.pdf]

Additional file 2

Supplementary Table S2. LNA GapmeR sequences designed to knock-down eRNAs

**LNA GapmeR**	**Sequence**
LNA_2S	T*A*G*A*A*C*T*T*G*G*T*T*G*A*T*G
LNA_2AS	T*G*A*G*A*A*C*A*G*A*G*T*T*G*A*A
LNA_4S	A*C*A*G*C*A*A*C*A*G*A*G*G*A*T*G
LNA_4AS	C*T*C*A*G*A*A*A*A*A*C*T*C*A*C*A
LNA_7S	C*A*T*A*A*C*G*A*G*G*T*C*T*G*G*A
LNA_7AS	G*G*C*T*G*C*A*A*A*G*A*A*A*T*T*G
LNA_NC	G*C*T*C*C*C*T*T*C*A*A*T*C*C*A*A

*Phosphorothioate backbone.
