# Supplementary material for: Enhancer RNA commits osteogenesis via microRNA-3129 expression in human bone marrow-derived mesenchymal stem cells
Source: Inflamm Regen. 2022 Sep 16;42:43. doi: 10.1186/s41232-022-00228-4 (PMC9479228; doi:10.1186/s41232-022-00228-4)
Supplement: Supplementary file 3 — Additional file 3: Supplementary Table S3. List of 45 genes predicted to be regulated by osteoblast-specific SEs based on the dbSUPER database. [file 41232_2022_228_MOESM3_ESM.pdf]

Additional file 3

Supplementary Table S3. List of 45 genes predicted to be regulated by osteoblast-specific SEs based on the dbSUPER database

Coding genes				Non-coding genes
BBS9	*LUM*	*RUFY1*	*UTP15*	*FLJ37453*
C2orf69	*LXN*	*SFTA1P*	*VIT*	*LINC00619*
CDK15	*MED23*	*SLC4A4*		*LOC145474*
CPQ	*MMP16*	*SMIM15*		*MIR132*
DDX60	*MTFR2*	*SNAPC1*		*MIR1469*
FBLN7	*NGF*	*SPCS3*		*MIR3129*
GOPC	*NPLOC4*	*TMEM241*		*NUDT6*
IFIT5	*NTN4*	*TMTC3*		
IMPAD1	*PDSS2*	*TPMT*		
INHBA-AS1	*PSMF1*	*TRIM34*		
KRTAP2-3	*PTPRQ*	*UBA52*		
LRRC49	*RASAL2-AS1*	*ULBP2*		
