# Supplementary material for: Enhancer RNA commits osteogenesis via microRNA-3129 expression in human bone marrow-derived mesenchymal stem cells
Source: Inflamm Regen. 2022 Sep 16;42:43. doi: 10.1186/s41232-022-00228-4 (PMC9479228; doi:10.1186/s41232-022-00228-4)
Supplement: Supplementary file 4 — Additional file 4: Supplementary Figure S1. miR-3129 gene expression pattern during adipogenesis and chondrogenesis of hBMSCs. Expression of miR-3129 was examined on Day 1, 7, 14, and 21 during adipogenesis (A) and chondrogenesis (B) of hBMSCs. The levels of miRNA expression were quantified by qPCR. The amounts of the miRNA transcript was expressed relative to the amount of U6sn transcript. Data are expressed as mean ± SD from three independent experiments (each n=3 in A and B). [file 41232_2022_228_MOESM4_ESM.pdf]

Additional file 4

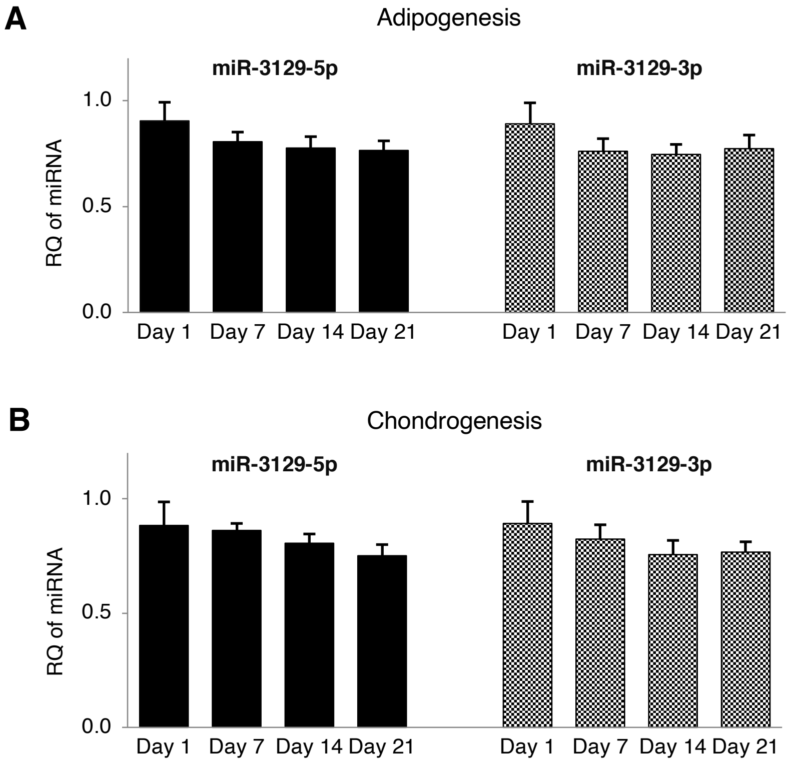

Supplementary Figure S1. *miR-3129* gene expression pattern during adipogenesis and chondrogenesis of hBMSCs. Expression of miR-3129 was examined on Day 1, 7, 14, and 21 during adipogenesis (**A**) and chondrogenesis (**B**) of hBMSCs. The levels of miRNA expression were quantified by qPCR. The amounts of the miRNA transcript was expressed relative to the amount of *U6sn* transcript. Data are expressed as mean ± SD from three independent experiments (each n=3 in **A** and **B**).
