# Supplementary material for: Enhancer RNA commits osteogenesis via microRNA-3129 expression in human bone marrow-derived mesenchymal stem cells
Source: Inflamm Regen. 2022 Sep 16;42:43. doi: 10.1186/s41232-022-00228-4 (PMC9479228; doi:10.1186/s41232-022-00228-4)
Supplement: Supplementary file 5 — Additional file 5: Supplementary Figure S2. Expression profiling of other putative target genes for miR-3129-5p after overexpression and knock-down of miR-3129 in hBMSCs. (A and B) At 48h after transfection of miR-3129-5p and -3p mimics (A) and -5p and -3p inhibitors (B), the levels of miR-3129-5p putative target gene expression were quantified by qPCR. The amounts of the mRNA transcript were expressed relative to the amount of GAPDH transcript. Data are expressed as mean ± SD from three independent experiments (each n=3 in A and B). m, mimic; in, inhibitor; NC, negative control. [file 41232_2022_228_MOESM5_ESM.pdf]

Additional file 5

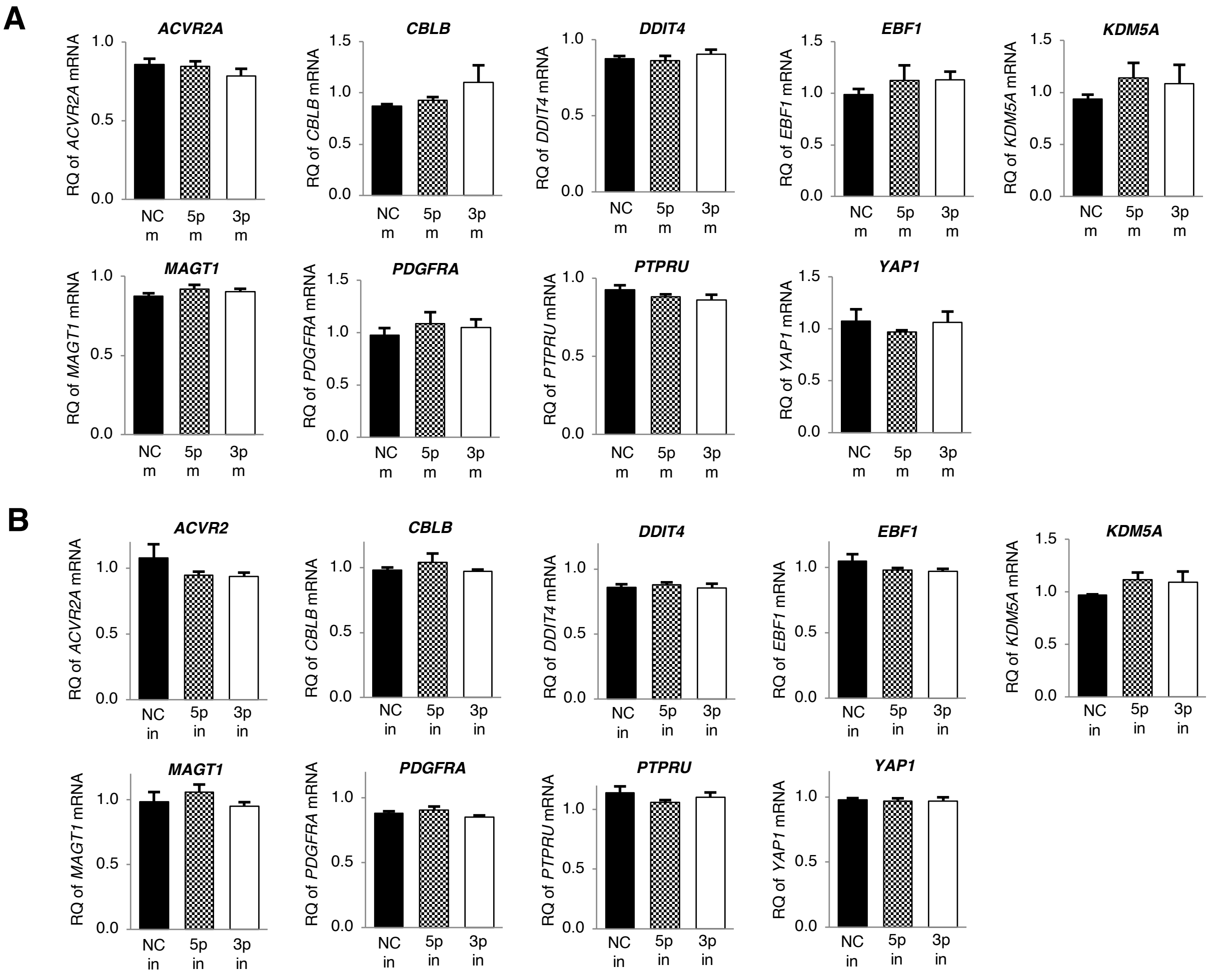

Supplementary Figure S2. Expression profiling of other putative target genes for miR-3129-5p after overexpression and knock-down of miR-3129 in hBMSCs. (A, B) At 48h after transfection of miR-3129-5p and -3p mimics (**A**) and -5p and -3p inhibitors (**B**), the levels of miR-3129-5p putative target gene expression were quantified by qPCR. The amounts of the mRNA transcript were expressed relative to the amount of *GAPDH* transcript. Data are expressed as mean ± SD from three independent experiments (each n=3 in **A** and **B**). m, mimic; in, inhibitor; NC, negative control.
