# Supplementary material for: Enhancer RNA commits osteogenesis via microRNA-3129 expression in human bone marrow-derived mesenchymal stem cells
Source: Inflamm Regen. 2022 Sep 16;42:43. doi: 10.1186/s41232-022-00228-4 (PMC9479228; doi:10.1186/s41232-022-00228-4)
Supplement: Supplementary file 6 — Additional file 6: Supplementary Figure S3. Knock-down of eRNA_2S enhanced SLC7A11 gene expression in hBMSCs. (A and B) At 24h after transfection of the indicated sense (A) and antisense (B) LNA GapmeRs, the levels of SLC7A11 expression were quantified by qPCR. The amounts of the SLC7A11 transcript were expressed relative to the amount of GAPDH transcript. Data are expressed as mean ± SD from three independent experiments (each n=3 in A and B). ***p<0.001 versus NC, by Student’s t-test. LNA, Locked Nucleic Acid; S, sense; AS, antisense; NC, LNA negative control. [file 41232_2022_228_MOESM6_ESM.pdf]

Additional file 6

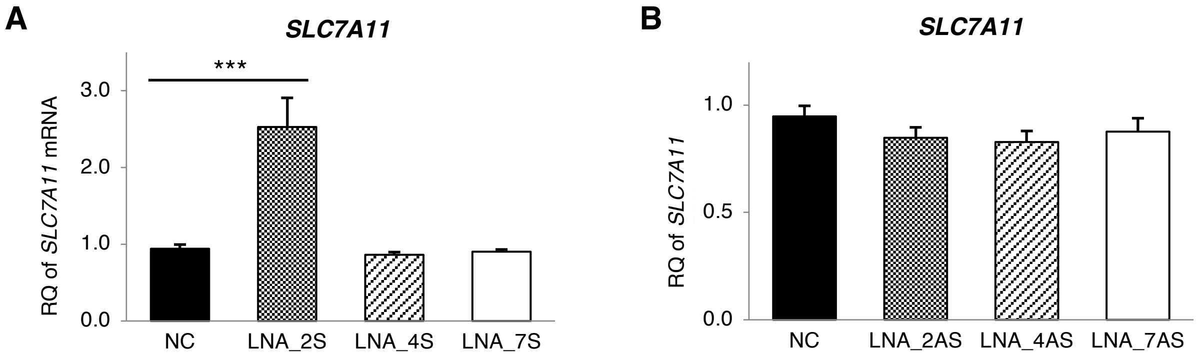

Supplementary Figure S3. Knock-down of eRNA_2S enhanced *SLC7A11* gene expression in hBMSCs. (A, B) At 24h after transfection of the indicated sense (**A**) and antisense (**B**) LNA GapmeRs, the levels of *SLC7A11* expression were quantified by qPCR. The amounts of the *SLC7A11* transcript were expressed relative to the amount of *GAPDH* transcript. Data are expressed as mean ± SD from three independent experiments (each n=3 in **A** and **B**). ***p<0.001 versus NC, by Student's t-test. LNA, Locked Nucleic Acid; S, sense; AS, antisense; NC, LNA negative control.
