# Supplementary material for: Enhancer RNA commits osteogenesis via microRNA-3129 expression in human bone marrow-derived mesenchymal stem cells
Source: Inflamm Regen. 2022 Sep 16;42:43. doi: 10.1186/s41232-022-00228-4 (PMC9479228; doi:10.1186/s41232-022-00228-4)
Supplement: Supplementary file 7 — Additional file 7: Supplementary Figure S4. All full-length images of Western blotting data. Uncropped full-length images are shown on Figure S4, and the images cropped from the data (n=1) on the Figure S4 are shown on the right side of Fig. 6B. Western blotting was performed one time. [file 41232_2022_228_MOESM7_ESM.pdf]

Additional file 7

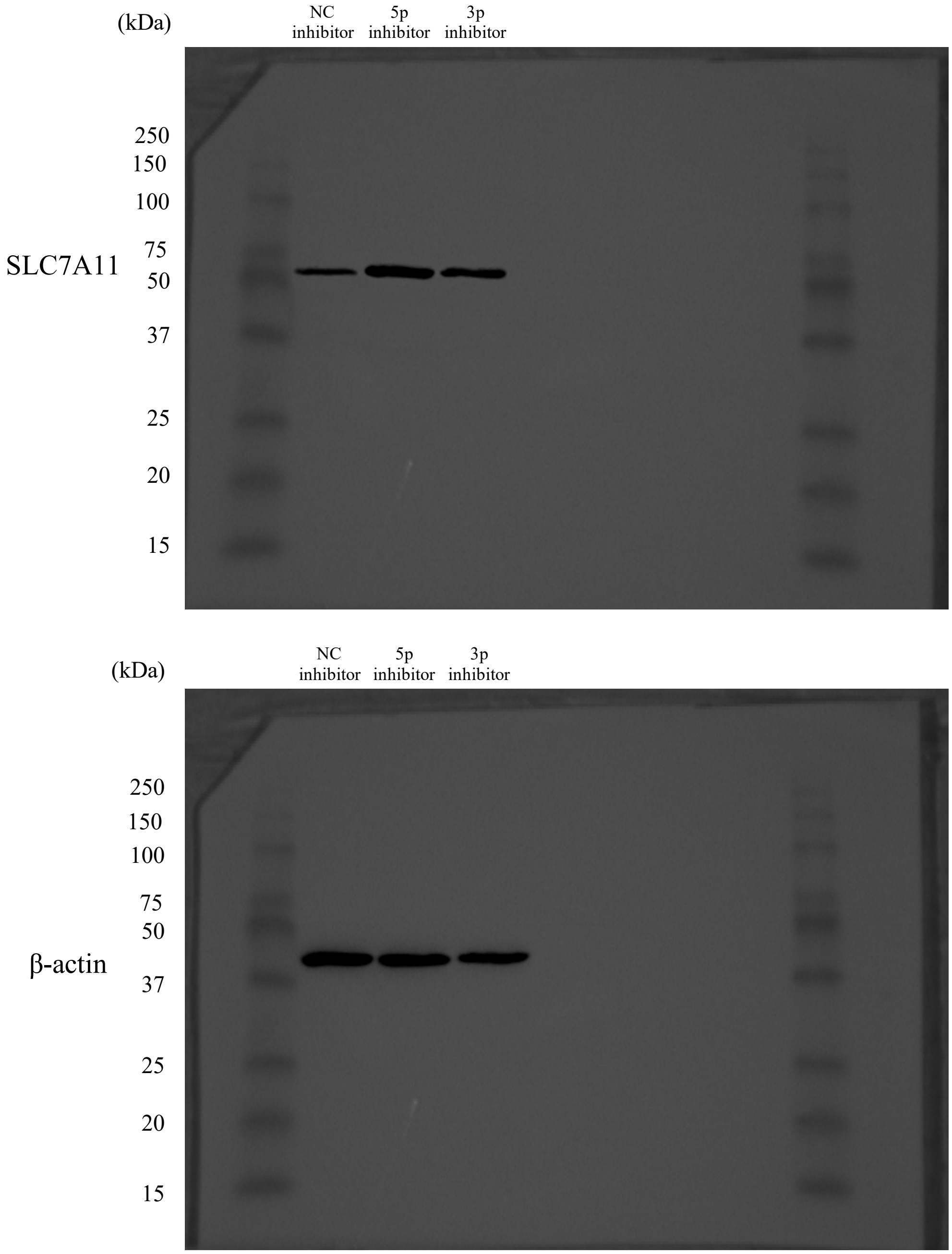

Supplementary Figure S4. All full-length images of Western blotting data. Uncropped full-length images are shown on Figure S4, and the images cropped from the data (n=1) on the Figure S4 are shown on the right side of Figure 6B. Western blotting was performed one time.
