# Supplementary material for: Enhancer RNA commits osteogenesis via microRNA-3129 expression in human bone marrow-derived mesenchymal stem cells
Source: Inflamm Regen. 2022 Sep 16;42:43. doi: 10.1186/s41232-022-00228-4 (PMC9479228; doi:10.1186/s41232-022-00228-4)
Supplement: Supplementary file 8 — Additional file 8: Supplementary Figure S5. A model of the expression mechanism for miR-3129 involved in MSC osteogenesis. An eRNA is expressed during the early stage of MSC osteoblast differentiation, and then induces miR-3129 expression via SE activation. The miRNA targets the mRNA of SLC7A11, which encodes a suppressor of osteogenesis, followed by down-regulation of SLC7A11 expression. This event contributes to MSC osteogenesis. BMSC, bone marrow-derived mesenchymal stem cell; RNA Pol II, RNA polymerase II; SE, super-enhancer; TF, transcription factor; TSS, transcription start site. [file 41232_2022_228_MOESM8_ESM.pdf]

Additional file 8

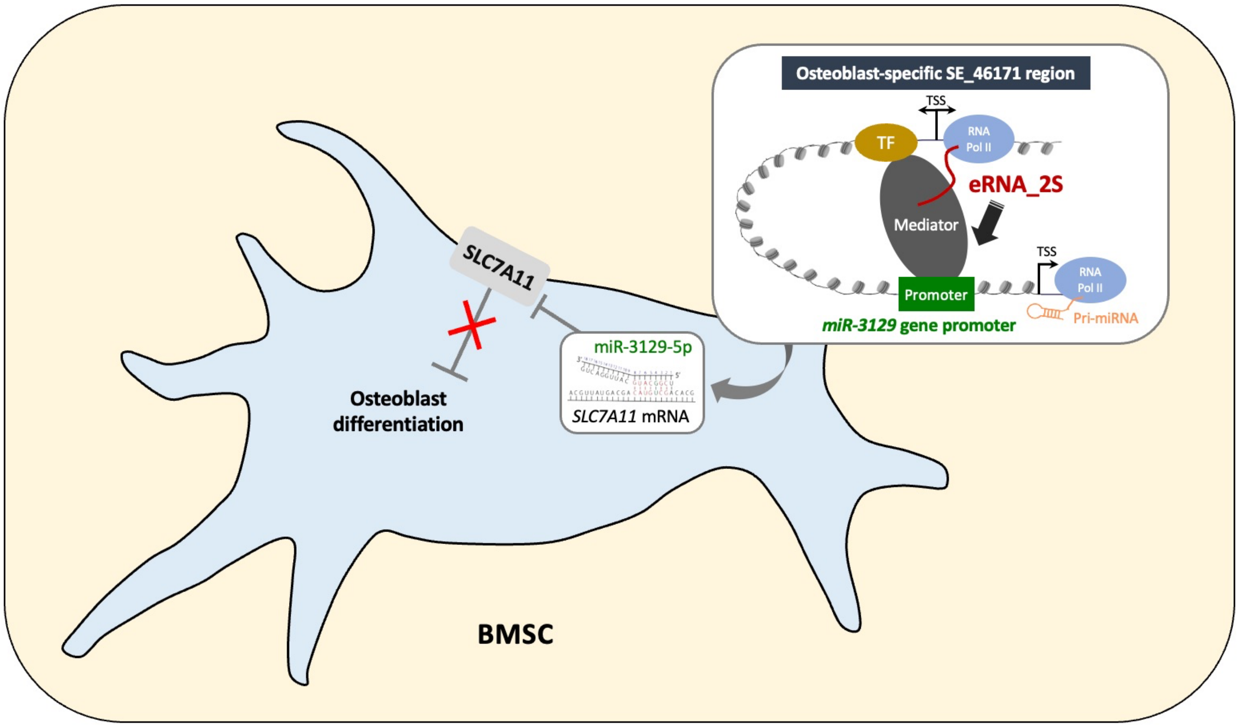

Supplementary Figure S5. A model of the expression mechanism for miR-3129 involved in MSC osteogenesis. An eRNA is expressed during the early stage of MSC osteoblast differentiation, and then induces miR-3129 expression via SE activation. The miRNA targets the mRNA of *SLC7A11*, which encodes a suppressor of osteogenesis, followed by down-regulation of *SLC7A11* expression. This event contributes to MSC osteogenesis. BMSC, bone marrow-derived mesenchymal stem cell; RNA Pol II, RNA polymerase II; SE, super-enhancer; TF, transcription factor; TSS, transcription start site.
